# Microbiota from *Litopenaeus vannamei*: digestive tract microbial community of Pacific white shrimp (*Litopenaeus vannamei*)

**DOI:** 10.1186/2193-1801-3-280

**Published:** 2014-06-02

**Authors:** Jaqueline Tuyub Tzuc, Diana Rendíz Escalante, Rafael Rojas Herrera, Gabriela Gaxiola Cortés, Maria Leticia Arena Ortiz

**Affiliations:** Unidad Multidisciplinaria de Docencia e Investigación (UMDI), Universidad Nacional Autónoma de México (UNAM), Puerto de Abrigo s/n, Sisal, Yucatan Mexico; Campus de Ingenierías y Ciencias Exactas, Universidad Autónoma de Yucatán. Facultad de Ingeniería Química, Periférico Norte Kilómetro 33.5, Tablaje Catastral 13615, Col. Chuburná de Hidalgo Inn., C.P. 97203 Mérida, Yucatán México

**Keywords:** Shrimp, Microbiota, Digestion, Enzymatic activity, 16S

## Abstract

Bacteria capable of producing different extracellular enzymes of potential relevance in digestive processes were isolated from the stomach, hepatopancreas and intestine of Pacific white shrimp *Litopenaeus vannamei.* A total of 64 strains with proteolytic activity were isolated and grouped into 16 clusters based on morphological characteristics: 4 groups were isolated from the intestine; 5 from the hepatopancreas; and 7 from the stomach. Molecular methods (16S rRNA gene amplification and sequencing) and phenotypic criteria (Gram stain, catalase and oxidase tests, cell and colony morphology) were used to identify strains, which corresponded to *Pseudoalteromonas* and *Vibrio* genera. These genera are reported to form part of the digestive tract microbial community in shrimp. Both genera were isolated from all three tested tissues*.* One member of each morphologic group was selected for analysis of the presence of amylases, lipases/esterases and chitinases. Most of the strains had all the tested enzymes, indicating that the *L. vannamei* digestive tract microbiotic flora includes groups which have the potential to contribute to the degradation of dietary components.

## Introduction

White shrimp *Litopenaeus vannamei* is one of the principal crustacean species cultured worldwide. It has been widely studied, although further research is still needed on its physiology and metabolism under specific environmental conditions, as well as its interactions with other organisms.

Gastrointestinal tract microbiota development is a gradual process beginning at birth. In terrestrial animals, maternal microbiota is the initial bacterial colonization source. In aquatic animals, however, this is determined by contact with the surrounding environment, and is influenced by feed intake, hormone secretion, nutrient absorption, and the appearance of proteins and digestive enzymes. Facultative anaerobic strains initially dominate in the intestine after which variation in microbiota population depends on diet, age, geographic location, medical treatment and overall organism condition (Cahill 1990; Isolauri et al. 2001). Gastrointestinal tract colonization by microbiota in white shrimp occurs during the nauplius 5 stage, when an anal pore begins "anal drinking" movements; this happens before the mouth opens to the exterior environment and normal colonization begins (Simoes et al. 2002).

Throughout the life of an organism, its microbiota provide metabolic, trophic and protective functions. Their metabolic functions are aimed at assisting in digestive processes and nutrient absorption to provide energy to the organism. Their trophic functions promote cell growth and differentiation, as well as stimulating of the immune system. Their protective functions are present from birth since they act as the first line of defense against pathogenic, exogenous or opportunist microorganisms, creating a barrier effect (Isolauri et al. 2001).

The microbial community of a cultured species can play an important role in aquaculture because it can affect host growth and survival. In marine organisms, gut microbes are associated with digestive enzyme production (Sugita et al. 1997; Ramirez and Dixon 2003; Izvekova 2006; Kar and Ghosh 2008), competitive exclusion of pathogenic bacteria, and generation of essential elements for host metabolism. However, these studies have largely been done with fish species, with little data available on the relevance of gut microbiota in the digestive processes of shrimp. In one such study of krill *Meganyctiphaunes norvegica* (Donachie et al. 1995), both the stomach and hepatopancreas were found to contain saprophytic bacteria related to enzymatic activity. Antiobiotic treatment for 72 hours reduced bacteria concentration and cellulosic, chitinolytic and laminarase activity compared to a control. This diminished bacterial concentration was correlated to the decreases in enzyme activities, suggesting that these bacteria were in a commensalist relationship with the enzymes.

Shrimp species of the family Penaeidae are omnivores, although some do have carnivorous or herbivorous tendencies. They have broad biochemical capacities to use diverse energetic sources as elements in their diet (Rosas and Carrillo 2006). Pacific white shrimp *L. vannamei* prefers protein over carbohydrates as an energy source, meaning it has rather high dietary protein requirements (Tacon 1989; García-Galano 2006). However, it also use carbohydrates, mainly as a direct source of metabolic energy, as a substratum for chitin synthesis and for nucleic acids synthesis (Gaxiola et al. 2006). In addition to functioning as an energy source in *L. vannamei*, lipids are essential elements in cell structure and function, steroid hormone precursors, intercellular mediators, and are vital to nauplii growth, maturity and production (Rosas and Carrillo 2006).

Using culture-independent methods such as DGGE (Avilés-Gómez 2011), our research group has studied bacterial flora composition in the digestive tract of shrimp at different stages, when fasting, when given feed with and without antibiotics, in juveniles, adults, males and females. The objective of the present study was to isolate and identify bacteria from the stomach, hepatopancreas and intestine of *L. vannamei*. Bacteria in these organs are often capable of producing different extracellular enzymes of potential relevance in digestive processes due to their degradation of the principal components in this species’ diet. Understanding the enzymatic capabilities of *L. vannamei* digestive system bacteria is important for creating a data baseline from which further research can explore their possible application as probiotics. It can also help in optimizing feed formulation to reduce production costs and improve feed digestibility in shrimp culture systems.

## Methods

### Biological material

Ten individuals of *Litopenaeus vannamei* were collected from culture ponds at the UMDI-Sisal, and transported live to the laboratory. These had been fed a commercial pellet feed and were grown using a Biofloc type culture system. They were dissected immediately under sterile conditions. The hepatopancreas, stomach and intestine of each were removed, and each organ run through a series of dilutions, beginning at a 1:10 ratio (1 g tissue/9 ml 2% NaCl sterile solution) and continuing progressively to a 1 × 10^-5^ dilution.

### Isolation of bacteria with proteolytic activity

Using the plate extension technique, dilutions were inoculated into casein-enriched marine agar (Difco), with two replicates per dilution. The inoculated dishes were incubated for 24 hours at 27°C ± 1 and strains selected that exhibited a transparent halo, indicating casein hydrolysis. Because similar phenotypes may be different strains, additionally three colonies sharing similar morphologic characteristics were selected per each selected colony. Each colony was inoculated into marine agar (Difco) in Petri dishes and incubated for 24 hours at 27°C ± 1. Using the plate streak method (24 h at 27°C ± 1), re-inoculation of the selected colonies continued until pure strains were obtained. These pure strains were analyzed with catalase and oxidase biochemical tests, and stain characteristics identified by the Gram technique. Each strain was placed in glycerol and stored at -80°C until further analysis.

### Molecular identification of isolated strains

The isolated strains were recovered from glycerol storage and their DNA extracted by thermal shock. Bacterial rRNA 16S sequences were amplified using the 16SS (5′-AGAGTTTGATCCTGGCTCAG-3′) (Edwards et al. 1989) and 16SR (5′-CGGGAACGTATTCACCG-3′) (Strom et al. 2002) primers. Amplification was done following a thermal cycle: 1 cycle at 95°C/1 min; 30 cycles at 95°C/1 min, 45°C/45 s and 72°C/1 min; 1 cycle at 72°C/5 min; and 1 cycle at 4°C/∞. Final PCR reaction volume was 25 μl, which contained 5 μl 5X colorless Gotaq Flexi buffer; 1.5 μl MgCl_2_; 0.5 μl dNTPs; 1 μl of each primer; 0.2 μl Gotaq Hot Start polymerase; 13.8 μl nuclease-free water; and 2 μl DNA. Product quality was verified by electrophoresis on 1% agarose gel stained with ethidium bromide, and the target amplicon cut out and purified. Amplicons were purified using a commercial kit (Wizard sv gel and PCR clean-up system, Promega) following manufacturer’s instructions. Final products were analyzed in 1% agarose gel stained with ethidium bromide, and sequencing done at the Biotechnology Institute (Instituto de Biotecnología - IBT, UNAM). A sequence homology search for the test sequences was done using Nucleotide Blast versus Genebank and RDP (Ribosomal Database Project) data. Results were validated with a phylogenetic analysis using the Winclada program and the Ratchet method (Island Hopper) with a *Micrococcus luteus* strain as external group.

### Other enzymatic activities

To determine if the isolated strains produced other types of extracellular enzymes, one representative group was selected from each morphological group and tested for amylase, chitinase and lipase/esterase activities.

### Amylases

Amylase activity was detected using Peptone agar and Von Hofsten and Malmquist Medium B (Atlas 2005) enriched with 2% starch and incubated for 24 hours at 27°C ± 1. To determine amylolytic activity, lugol was added to the surface of the medium containing the colonies, and those clearly exhibiting a halo deemed positive.

### Chitinases

Strains were inoculated into chitin agar (Atlas 2005) enriched with 2% NaCl and incubated at 27°C ± 1, and the strain capacity to grow in this medium observed.

### Lipases/esterases

Activity for these enzymes was tested using a medium described by Sánchez-Porro (Sánchez-Porro 2005), modified by changing the concentration of the salts to 10% and that of NaCl to 2% . Each strain was inoculated into this medium and incubated at 27°C ± 1 until a zone of precipitation formed around the colonies as a consequence of the reaction between CaCl_2_ and the fatty acids released by hydrolysis of Tween 80.

## Results

### Isolation of strains with proteolytic activity

A total of 64 strains with proteolytic activity against casein were isolated from the digestive tract of *L. vannamei*. These trains clustered into 16 groups based on morphological characteristics: 4 groups from the intestine, 5 from the hepatopancreas and 7 from the stomach.

### Identification of isolated strains

**Figure 1:**
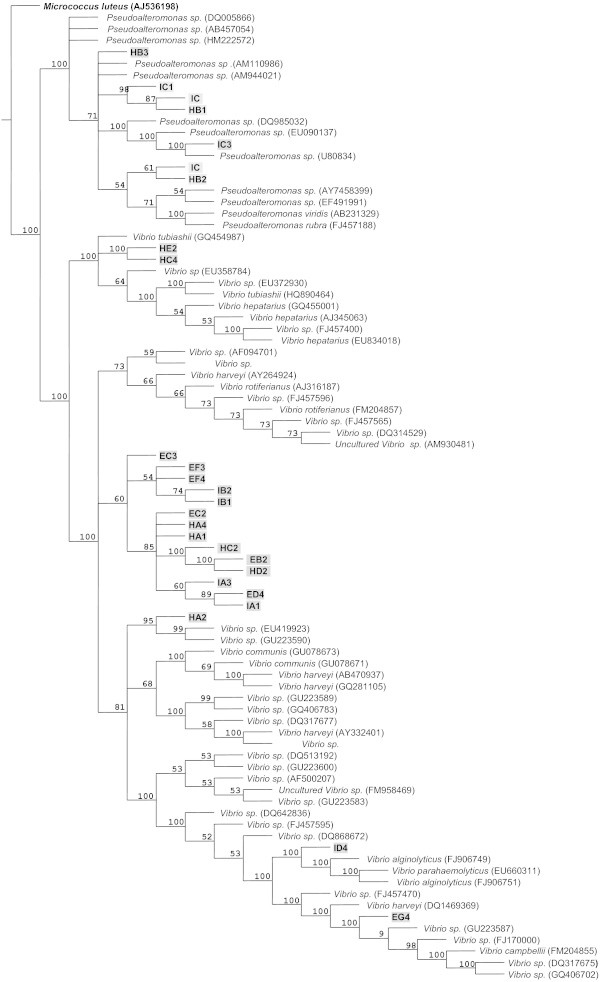
**Phylogenetic tree based on RNAr 16S gene sequences (Ratchet method, Length: 317, consistency index: 70%, retention index: 95%).** We show the phylogenetic relation of the studied strains with other groups. *Micrococcus luteus* AJ536198 was used as an external group.

**Figure 2:**
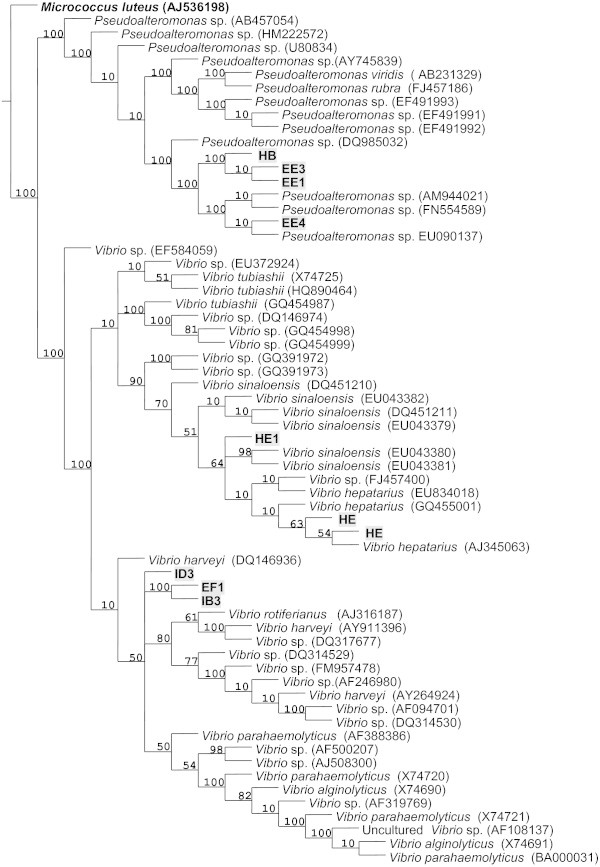
**Phylogenetic tree based on RNAr 16S gene sequences (Ratchet method, Length: 307, consistency index: 60%, retention index: 91%).** We show the phylogenetic relation of the studied strains with other groups. *Micrococcus luteus* AJ536198 was used as an external group.

Using the 16SS and 16SR primers, a 1300 bp amplicon for the rRNA 16S gen was isolated from the strains. Sequencing and homology queries in databases produced values ≥90% for maximum identity and E values near 0 for the Nucleotide Blast analysis. Sequence similarity and S-ab scores based on the RDP database had values near 1. The identified homologies correspond to strains belonging to species of the genera *Pseudoalteromonas* and *Vibrio* (Table 1). Results were consistent in both consulted databases. Dendrograms with high consistency and retention indices were produced with a phylogenetic analysis using the identified homologies (Figures 1 and 2). The phylogenetic results validated the biodata analysis results. All identified strains were Gram negative and had reacted positively to catalase and oxidase tests. This is consistent with the accepted general descriptions of *Pseudoalteromonas* and *Vibrio*.

**Table 1:** **Homologies of the isolated strains with 16S ribosomal DNA gene sequences in the RDP (Ribosomal Database Project) database**

Strain	Homology access no.	Description	Similarity	S-AB score
**EB2**	AY332401	*Vibrio harveyi*	0.997	0.988
	GU078671	*Vibrio communis*	0.997	0.992
	GU078673	*Vibrio communis*	0.997	0.992
**EC2**	EU419923	*Vibrio* sp.	0.995	0.992
	GU223590	*Vibrio* sp.	0.995	0.992
	GU078673	*Vibrio communis*	0.995	0.987
**EC3**	AJ316187	*Vibrio rotiferianus*	0.991	0.957
	AY264924	*Vibrio harveyi*	0.991	0.957
	EU419923	*Vibrio sp.*	0.991	0.963
**ED4**	GU223600	*Vibrio* sp.	0.993	0.961
	GU223583	*Vibrio* sp.	0.993	0.961
	DQ513192	*Vibrio* sp.	0.993	0.963
**EE1**	DQ985032	*Pseudoalteromonas* sp.	0.979	0.937
	EU090137	*Pseudoalteromonas* sp.	0.983	0.937
	AY745839	*Pseudoalteromonas* sp.	0.979	0.940
**EE3**	U80834	*Pseudoalteromonas* sp.	0.994	0.987
	DQ985032	*Pseudoalteromonas* sp.	0.994	0.987
	EU090137	*Pseudoalteromonas* sp.	0.994	0.987
**EE4**	EU090137	*Pseudoalteromonas* sp.	1.000	0.977
	DQ985032	*Pseudoalteromonas* sp.	1.000	0.977
	U80834	*Pseudoalteromonas* sp.	1.000	0.977
**EF1**	AF246980	*Vibrio* sp.	0.994	0.974
	AY264924	*Vibrio harveyi*	0.994	0.974
	FM957478	*Vibrio* sp.	0.994	0.983
**EF3**	AJ316187	*Vibrio rotiferianus*	0.997	0.991
	FJ457565	*Vibrio* sp.	0.998	0.991
	FM204857	*Vibrio rotiferianus*	0.998	0.991
**EF4**	AJ316187	*Vibrio rotiferianus*	0.993	0.977
	AY264924	*Vibrio harveyi*	0.993	0.977
	FJ457565	*Vibrio* sp.	0.995	0.977
**EG4**	GU223587	*Vibrio* sp.	0.997	0.992
	GQ406702	*Vibrio* sp.	0.997	0.992
	FM204855	*Vibrio campbellii*	0.999	0.992

### Amylase

Of the isolated *Vibrio* strains, amylase activity was observed in one strain from the hepatopancreas (HD2), two from the stomach (EF3, EG4) and one from the intestine (IA1). No activity was observed in one strain from the stomach (ED4) and one from the intestine (ID4), and the remaining *Vibrio* strains exhibited low activity (Table 2). None of the *Pseudoalteromonas* strains (i.e. EE1, HB1, and IC1) exhibited amylase activity (Table 2).

**Table 2:** ***Pseudoalteromonas*** **and** ***Vibrio*** **strains isolated from the stomach, hepatopancreas and intestinal tissues of** ***L. vannamei*** , and their extracellular enzymatic production (A = Amylases, L = Lipases/Esterases, Ch = Chitinases, black dots [●] = enzymatic activity detected, (*) = no activity detected)

		Stomach				Hepato				Intest		
		A	L	Ch		A	L	Ch		A	L	Ch
*Pseudoalteromonas*	EE1		●	●	HB1		●	●	IC1	*	●	●
	EB2	●	●	●	HA1	●	●	●	IA1	●	●	●
	EC2	●	●	●	HC2	●	●	●	IB1	●	●	*
*Vibrio*	ED4	*	●	●	HD2	●	●	●	ID4	*	●	●
	EF3	●	●	●	HE2	●	●	●				
	EG4	●	●	●								

### Chitinase

All strains had chitinase activity, except the *Vibrio* strain (IB1) isolated from the intestine. However, activity was generally low in both genera (*Vibrio* and *Pseudoalteromonas*), particularly in HB1 (hepatopancreas), EE1 (stomach) and IC1 (intestine) (Table 2).

### Lipase/esterase

All tested strains (*Vibrio* and *Pseudoalteromonas*) exhibited lipase/esterase activity (Table 2). Two strains from the stomach (EE1, EF3), one from the hepatopancreas (HB1) and two from the intestine (IB1, IB4) had low or nearly absent activity while the remaining strains had high activity.

## Discussion

Bacterial strains were successfully isolated from the digestive tract of *L. vannamei*. Many of these were found to have the capacity for extracellular enzyme production, which may be relevant in the digestive processes of this species. Isolated strains belonged to the *Vibrio* and *Pseudoalteromonas* genera, both of which are normal flora in the shrimp digestive system, although *Vibrio* species are normally the most abundant (Gomez-Gil et al. 1998; Moss et al. 2000; Oxley et al. 2002; Esiobu and Yamazaki 2003; Liu et al. 2011). Strains from both genera were isolated from the three sampled tissues. This agrees with previous reports in which the genera *Aeromonas*, *Plesiomonas*, *Photobacterium*, *Pseudomonas*, *Pseudoalteromonas* and *Vibrio* were isolated and identified from different parts of the *Penaeus merguiensis* digestive tract (Oxley et al. 2002). *Pseudoalteromonas* is a common genus in the marine environment with about 30 species present in sea water, algae or marine invertebrates (Ivanova et al. 2002; Holmström et al. 2002). These species produce several chemical compounds, including amylases (Gavrilovic et al. 1982; Aghajari et al. 1998), β-galactosidases (Hoyoux et al. 2001), phospholipases (Cadman and Eichberg 1983), antimicrobial compounds (Holmström et al. 2002) and proteases (Holmström and Kjelleberg 1999; Venkateswaran and Dohmoto 2000). Some *Pseudoalteromonas* species have been described as pathogens (Ridgway et al. 2008; Wang 2011), while others have been used as probiotics in marine organisms, including shrimp (Le Moullac et al. 2003; Schulze et al. 2006; Doeschate and Coyne 2008). Similarly, *Vibrio* species are broadly distributed in marine environments and are associated with a wide variety of organisms. Many are known to be pathogens, some in shrimp (Sung et al. 2001; Hsu and Chen 2007), while others have been tested as probiotics (e.g. *V. alginolyticus*, *V. fluvialis* and *V. campbellii*) (Verschuere et al. 2000; Schulze et al. 2006; Balcázar et al. 2007).

Proteins, carbohydrates and lipids are among the principal components of the shrimp diet and consequently represent the highest weight percentage in feed formulations (Akiyama et al. 1991). Digestion in the shrimp gastrointestinal tract allows uptake of monomers such as amino acids, sugars and fatty acids (Nolasco et al. 2006). These monomers, as well as other free monomers already in the diet, represent the principal fraction to be assimilated and metabolized from any formulated or natural feed (Akiyama et al. 1991; Shiau 1997). Most of the identified strains produced all the tested enzymes (proteases, amylases, lipases/esterases and chitinases), indicating that *L. vannamei* bacterial flora includes some groups exhibiting multienzymatic activity. This agrees with (Dempsey and Kitting 1987), in which isolated bacteria from the digestive tract of *Penaeus aztecus* expressed for one or more enzyme types (lipases, amylases, chitinases and cellulases).

The multienzymatic capacity observed here suggests that the isolated strains have a high potential for degrading the principal dietary components of *L. vannamei*. However, this capability is restricted by digestive tract environmental conditions, which may or may not favor enzymatic production. Bacterial stress factors such as pH, oxygen levels and nutrient availability can affect the production of certain enzymes; for example, reports that variations between pH 4.4 and 10.5 affect the metalloprotease production of *Pseudoalteromonas atlantica*. Nonetheless, both *Pseudoalteromonas* and *Vibrio* include species capable of surviving and reproducing under digestive tract conditions in different aquatic species, including decapod crustaceans (Hoffman and Decho 2000).

*Pseudoalteromonas* species are adapted to wide ranges of temperature (10-37°C), salinity (1-10% NaCl) and pH (6-10, optimum 6 - 7) (Venkateswaran and Dohmoto 2000; Hoffman and Decho 2000). Some *Vibrio* species can grow within an even wider temperature range (4 – 40°C) and in similar salinities (1 to 9% NaCl) (Fujino et al. 1974; Hada et al. 1984; Thompson et al. 2003; Gómez-Gil et al. 2003; Gómez-Gil et al. 2008). Based on their ability to prosper under these conditions, it is quite possible that the microbiotic enzymatic activities identified here *in vitro* could also occur *in vivo*.

Several studies, principally with fish, have shown the active role that exogenic enzymes have in food digestion within the digestive tract. There is still some debate about the magnitude of the contribution of these enzymes to total enzymatic activity. Detection and characterization of protease enzymes in shrimp has involved several approaches, but enzyme purification has only been done in a few studies. In most studies, the presence of enzymes has been detected with raw extracts, using synthetic substrates or specific inhibitors for each of the tested enzymes (Carrillo and González 2000; Forrellat and Gaxiola 2006). This is also the case in studies addressing enzymes that degrade carbohydrates. As a result, it is still unclear whether enzymatic activity in the shrimp digestive tract is produced completely by the organism or is at least partially due to its bacterial flora.

The *Pseudoalteromonas* strains identified here (EE1, HB1, IC1) exhibited no amylase activity, although this activity has been identified in other *Pseudoalteromonas* species (e.g. *P. peptidolytica*, *P. aurantia*, *P. citrea*, *P. luteoviolacea* and *P. rubra*) (Gavrilovic et al. 1982; Venkateswaran and Dohmoto 2000). Amylase activity was observed in most of the *Vibrio* strains, except for ED4 and ID4. This activity has been reported for several *Vibrio* species, including *V. tubiashii*, *V. parahaemolyticus* and *V. sinaloensis* (Hada et al. 1984; Fujino et al. 1974; Gómez-Gil et al. 2008), as well as other strains not identified to the species level (León et al. 2000). Because the same temperature and pH values were used for all the identified strains, the differences observed in activity intensity may be related to the response of each bacterial strain to the experimental parameters.

Chitinase activity has been identified in the digestive tract of several aquatic species, including some penaeid shrimps (Goodrich and Morita 1977; Kono et al. 1990; Spindler-Barth et al. 1990). Several authors agree that chitinase levels in shrimp are sufficient to allow for significant digestion of dietary chitin (Kono et al. 1990). Chitinases are secreted endogenously, but their presence in the shrimp digestive tract has also been attributed to chitinolytic bacteria. This is not surprising since chitin is one of the most abundant biopolymers in nature and is used as a carbon source by several microorganisms (Hood and Meyers 1973; Dempsey and Kitting 1987; Connell et al. 1998). Chitinase enzymes were reported to be produced by bacteria from the digestive tract of *Litopenaeus setiferus,* and increases in chitinase activity were observed in shrimp fed a chitin-rich diet with a parallel rise in chitinolytic bacteria counts (Hood and Meyers 1973). Chitinolytic bacteria have also been reported in the digestive tracts of *Penaeus aztecus* (Dempsey and Kitting 1987). Dependence on bacteria for chitinase activity in which growth rates did not increase in *Penaeus monodon* juveniles fed chitin-enriched diets at 0%, 4%, 8%, 12% and 16%. Low numbers of chitinolytic microorganisms were found in the organisms’ digestive tracts. It was concluded that chitinase synthesis by the digestive gland rises at a low rate and therefore the organisms could only digest small amounts of dietary chitin in the absence of bacterial chitinase production (Fox 1993).

Chitinase production has been detected in *Vibrio* species such as *V. vulnificus*, *V. harveyi* and *V. parahaemoliticus* (Connell et al. 1998). The same could be expected of *Pseudoalteromonas* species, an exclusively marine genus. However, the *Pseudoalteromonas* strains identified here had the lowest chitinase activity among the identified strains, and in a study of several species chitinase activity was only identified in *P. citrea* (Venkateswaran and Dohmoto 2000).

Very few studies have reported the presence of lipase in decapods. These include a study in which lipase activity was found in the digestive gland of *L. vannamei* using Beta-naphthyl nanonate as a substrate (Moss et al. 2001), and another in which lipolytic activity was attributed to non-specific esterases (Carrillo and González 2000). In the present study, all tested strains from the *L. vannamei* digestive tract exhibited lipase/esterase activity, indicating that lipase/esterase production is very common in the analyzed genera. This agrees with previous studies reporting lipase/esterase production in *Vibrio* and *Pseudoalteromonas* species (Emod et al. 1983; Hada et al. 1984; León et al. 2000; Venkateswaran and Dohmoto 2000; Gómez-Gil et al. 2008). Shrimp have low lipid requirements and consequently low lipase activity, meaning that diets containing high lipid levels (>10%) are not fully exploited by these organisms (Lista and Velásquez 2003). The lipid degrading capacity of some bacteria could therefore be used to increase lipid assimilation in cultured shrimp species such as *L. vannamei.*

*Vibrio* and *Pseudoalteromonas* species have been used as probiotics (Verschuere et al. 2000; Schulze et al. 2006; Balcázar et al. 2007). For a bacteria to be considered probiotic it must be capable of surviving passage through the gastrointestinal tract. It must then colonize the host digestive system, either by adhering to the mucous membrane surface or the intestinal epithelia. Finally, it must be able to produce inhibitory or antagonist metabolites against undesirable native flora, and reproduce (Thompson et al. 1999; Verschuere et al. 2000; Ziaei-Nejad et al. 2006). *Pseudoalteromonas* species are promising in this sense because they have been found in association with marine organisms and have the capacity to produce a wide range of compounds with antimicrobial activity. This allows them to compete with other microorganisms for nutrients and colonizing surfaces, thus reducing pathogen adherence (Holmström and Kjelleberg 1999). The present results show that the identified strains produce diverse exo-enzymes. This suggests they may be potential probiotics, with the distinct advantage that they are endemic to the *L. vannamei* digestive system.

The data reported here on the enzyme producing capabilities of shrimp gut bacteria can form the basis for future research aimed at optimizing shrimp culture practices. Specific studies are needed on the probiotic potential of the identified bacterial species and on developing shrimp feed formulations that provide maximum growth performance considering the organism’s gastrointestinal microbiota. Future developments in these areas could help to reduce production costs and augment feed digestibility.
